# Superficial radiotherapy as a treatment alternative for recurrent conjunctival squamous cell carcinoma: a case study

**DOI:** 10.1002/jmrs.274

**Published:** 2018-03-25

**Authors:** Amanda Edgar, Gretel Crutchfield, Nigel Anderson

**Affiliations:** ^1^ Department of Radiation Oncology and Cancer Imaging Peter MacCallum Cancer Centre Melbourne Victoria Australia

**Keywords:** Oncology, ophthalmology, radiation oncology, radiation therapy treatment, superficial x‐ray therapy (SXRT)

## Abstract

This case study discusses the use of superficial radiotherapy (SXRT) in the treatment of recurrent conjunctival squamous cell carcinoma (SCC). Conjunctival SCC is often an aggressive cancer, with surgery the current standard of care. There is currently limited literature on alternative treatment options to treat conjunctival SCC recurrences that enable ocular function preservation. Furthermore, the use of SXRT in this setting is not well‐reported. Technical feasibility, practical limitations and potential side effects of SXRT (in comparison to other treatment options) are discussed in this case study. This case describes a 62 years old male with limited treatment options following multiple recurrences of conjunctival SCC. He was prescribed a therapeutic SXRT dose of 48.4 Gy in 22 fractions (5 fractions/week). At 6‐month follow‐up, there was no evidence of residual or recurrent disease, or any significant objective or patient reported treatment induced side effects. This case study provides preliminary evidence for the potential application of SXRT for conjunctival SCC. The benefits reported in this case study warrant further investigation of the applicability of SXRT in a larger patient cohort, with the potential to provide patients with a less invasive treatment alternative for recurrent conjunctival SCC.

## Introduction

Squamous cell carcinoma (SCC) is a common malignancy of the ocular surface, with an incidence of three and a half cases per 100,000 Australians, of which 79% of cases are male (mean age of 61 years).^1^, ^2^ Associated risk factors such as human immunodeficiency virus, human pappiloma virus, ultraviolet exposure and immunosuppression are known to increase rates of incidence further.^1^, ^3^ A SCC of the conjunctiva is a sight threatening and potentially life threatening diagnosis as it can invade the anterior chamber of the eye and orbital septum or form distant metastasis.^3^ For this common and sight threatening condition there are a number of treatment options available, though all demonstrate high rates of recurrence.^4^

Surgical excision of the lesion is the most common intervention used to treat SCC of the conjunctiva. The recurrence rates after surgical excision are 30–40%.^5^ An adjunctive to surgical excision is cryotherapy, as a method of killing residual tumour cells.^6^ In these cases there is a reported recurrence rate of 22% that is independent of the stage of SCC, with 70% of recurrences occurring within the first 12 months post‐treatment.^7^, ^8^ These high rates of recurrence make it necessary to perform strict follow‐up of patients who receive treatment for SCC of the conjunctiva. For patients who are diagnosed with recurrent SCC of the conjunctiva there is limited clinical evidence to support one treatment alternative over another. The lack of evidence is an issue as recurrent SCC of the conjunctiva is more aggressive, growing and invading local structures more rapidly, increasing the need for successful treatment.^9^ In these cases, when local control is unsuccessful, the eye is removed surgically via a technique called enucleation. This will reduce patients' quality of life and significantly impact their visual function.^9^ Therefore, the development of a more cosmetically acceptable and effective treatment for recurrent SCC of the conjunctiva would be of benefit to patients diagnosed with this condition.

Superficial radiotherapy (SXRT) could provide a salvage treatment option for recurrent SCC of the conjunctiva. This treatment option aims to spare the ocular function of the eye and preserve cosmesis. A literature search did not reveal any previous studies detailing the use of SXRT for this diagnosis. This case study reports on, with informed consent from the patient, the use of SXRT treatment for recurrent SCC of the bulbar conjunctiva. All identifiable features, including photographs, have been deidentified.

## Case Presentation

A 62 years old male presented with recurrent SCC of the left nasal bulbar conjunctiva. The patient's previous medical history detailed multiple interventions at this site. Initially, the diagnosis of a left nasal pterygium (a wing‐shaped growth that starts on the conjunctiva and can spread across the limbus to the cornea affecting visual function) was made 3 years prior to the reported presentation, and was surgically removed with local excision.^10^ The lesion recurred 1‐year post‐surgery, and was subsequently managed surgically with a wide local excision. At this time, pathology revealed positive margins for SCC. A further recurrence 6 months later was treated with Plaque Therapy to a dose of 50 Gy in 5 fractions. Subsequently, the lesion was re‐excised with adjuvant cryotherapy 7 months later.

**Figure 1:**
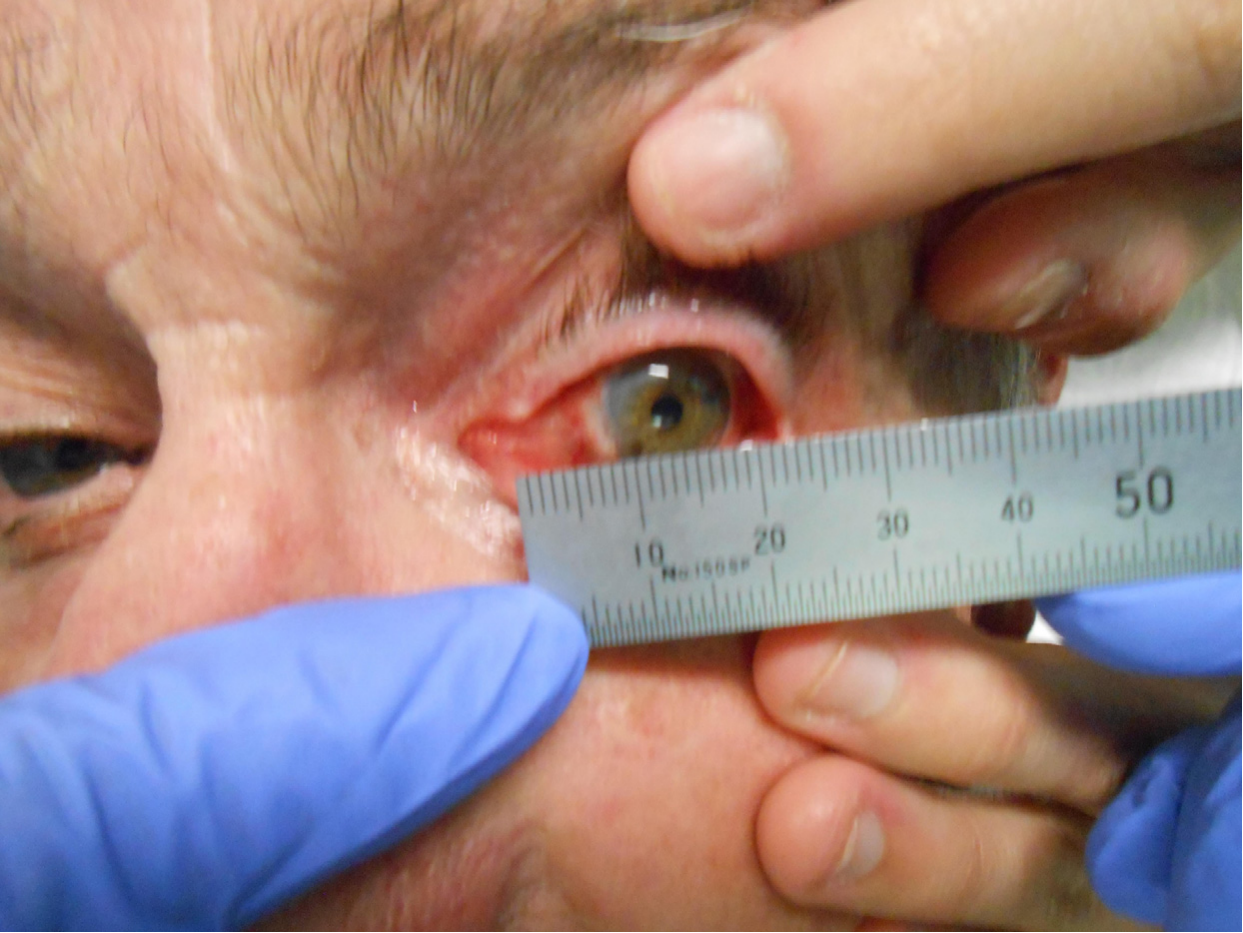
Original presentation of recurrent squamous cell carcinoma (SCC) on the medial ocular surface (Image used with patient permission, courtesy of Division of Radiation Oncology, Peter MacCallum Cancer Centre).

**Figure 2:**
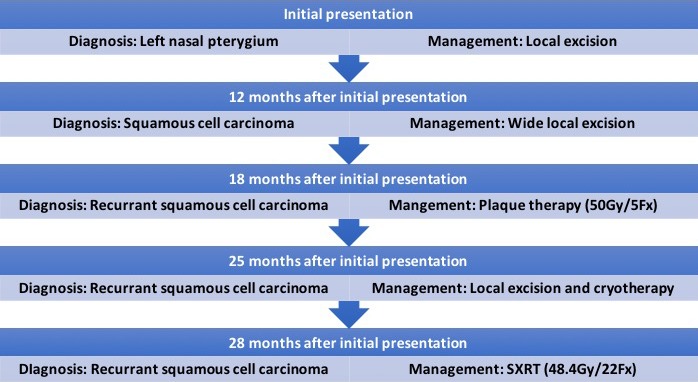
Flow chart depicting patient's previous treatment timelines prior to SXRT intervention.

On presentation 3 months post‐cryotherapy, a recurrent lesion (10 × 5 × 2 mm) was detected on the nasal bulbar conjunctiva (Fig. 1). The patient declined enucleation following this SCC recurrence. Subsequently, multidisciplinary consultation offered SXRT as an alternative treatment option (Fig. 2).

The patient also presented with a history of Crohns Colitis (treated with immunotherapy) and a previous excision of a non‐melanoma skin cancer from the right temple, and a subsequent skin graft of the area. The patient was on no other medications and had no allergies.

### SXRT treatment

**Figure 3:**
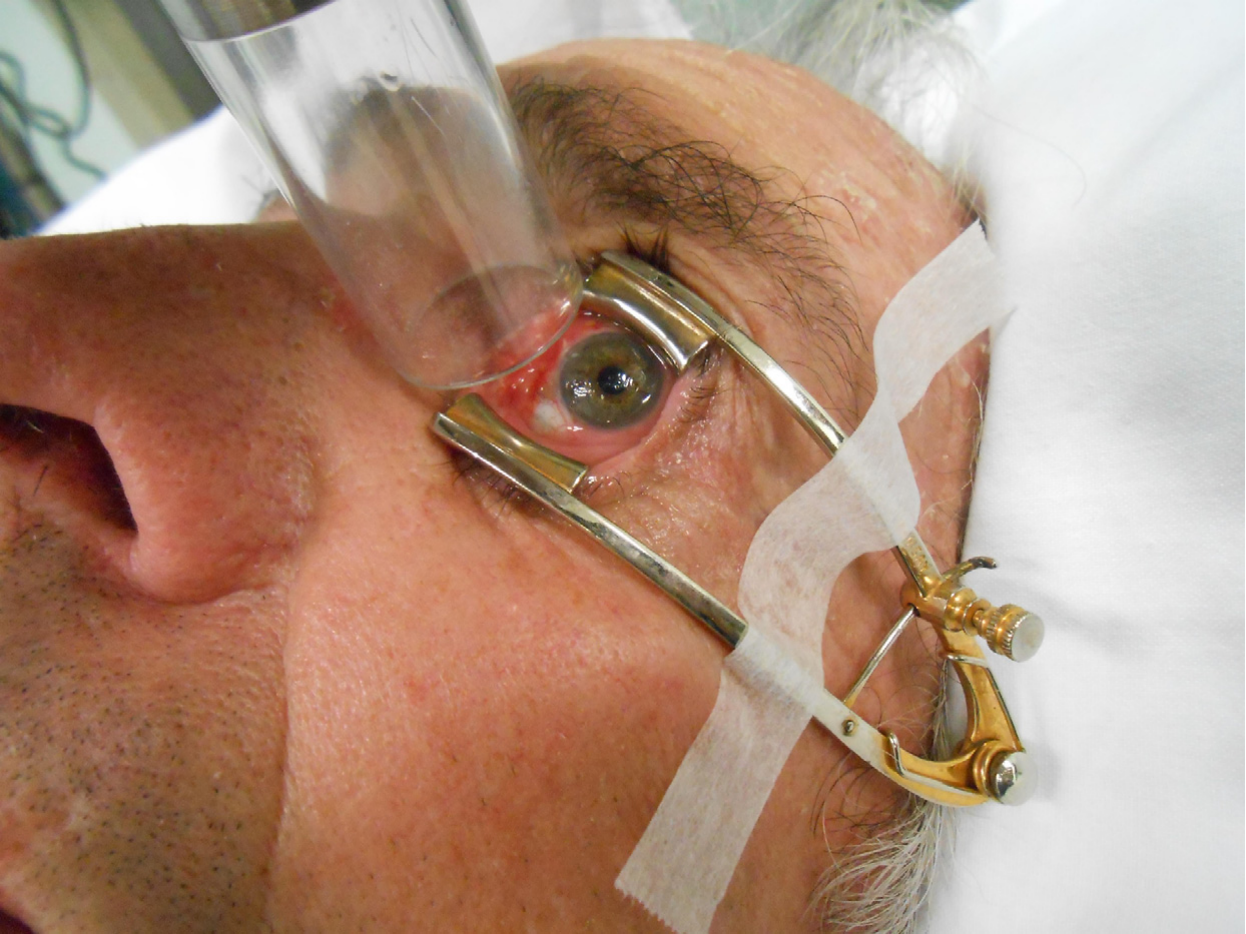
Eye retractors and applicator positioning for superficial radiotherapy treatment (SXRT) (Image used with patient permission, courtesy of Division of Radiation Oncology, Peter MacCallum Cancer Centre).

The patient was prescribed a dose of 48.4 Gy in 22 fractions, to a depth of 3 mm. This resulted in a skin surface dose of 54.4 Gy. A 2.0 cm diameter direct applicator was used with a 3 mm margin on the treatment region (Fig. 3). To allow for an acceptable treatment margin that encompases the target volume and accounts for set up variability, the left inner canthus and tear duct were included in the treatment field. A single en‐face beam of 2.0 mm Aluminium energy (2 mm Al) was used to meet the previously articulated dose prescription. The risk of ulceration, scleral perforation, ischemia and nasolacrimal duct obstruction was explained to the patient. This risk was further exacerbated in this case due to re‐irradiation of the previously treated area, and a particularly sensitive region post‐cryotherapy, surgery and plaque therapy. Topical anaesthesia (Tetracaine eyedrops) was administered to the left eye prior to retractor positioning, to ensure eyelids remained open throughout SXRT delivery. After positioning of the retractors the patient was instructed to maintain gaze on a target positioned to the left side of the room, and the eye was monitored throughout treatment delivery by video surveillance. Lubricating eye drops were prescribed for use prior to treatment administration to prevent drying of the ocular surface, and for the patients self‐use between fractions to relieve dry eye symptoms. Total set up and treatment time for this patient was approximately 20 min, with the retractors in place on average less than 10 min. The anaesthetic drops and ocular surface lubricants induced limited sensation and the patient found it mildly uncomfortable. As a precaution, the patient was informed to avoid contact with the anaesthetised eye and wear a protective patch for 60 min post‐treatment delivery daily.

## Results and Follow‐up

**Figure 4:**
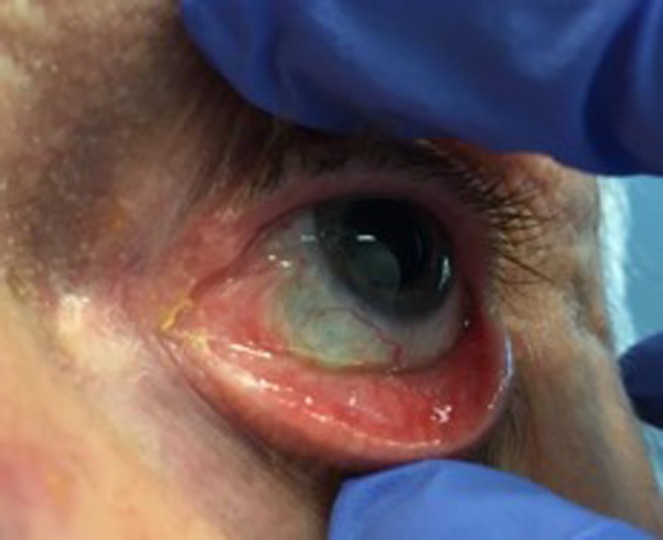
Surface of the eye at 6 months follow‐up. Some scarring present medially (Image used with patient permission, courtesy of Division of Radiation Oncology, Peter MacCallum Cancer Centre).

At 6 months follow‐up with the radiation oncologist, the patient reported mild irritation and symptoms of dry eye. Anterior examination demonstrated nasal bulbar conjunctival scarring (Fig. 4). There was no palpable lymphadenopathy within the neck, and no new skin lesions on the face or neck. The parotid and submandibular nodal regions were also clear of any recurrence. A magnetic resonance imaging (MRI) scan, requested by the ophthalmologist, confirmed the absence of recurrent disease.

## Discussion

Radiotherapy could provide an alternative for the treatment of SCC of the conjunctiva, resulting in improved cosmetic and functional outcomes. Radiotherapy can be in the form of plaque therapy, proton therapy, SXRT or electron beam irradiation.^11^, ^12^, ^13^ The advantage of radiotherapy is that it provides a localised treatment of the targeted area that aims for tumour control with additional avoidance of adjacent healthy tissue. When treating ocular neoplasia, radiotherapy enables ocular function preservation and improved cosmesis compared to the surgical removal of the eye by enucleation, that leaves the patient with one functional eye and monocular vision. The improved quality of life with treatment by radiotherapy compared to enucleation is discussed in literature.^9^ Melia et al. showed enucleation has a greater impact on activities of daily living, such as driving and peripheral vision, which reduce patients quality of life.^9^ This demonstrates the potential for using radiotherapy to treat recurrent SCC of the conjunctiva by providing tumour control and preserving visual function with minimal side effects. This case provides preliminary evidence SXRT may be a suitable treatment option for recurrent SCC of the conjunctiva.

Whilst electron beam radiotherapy is an option when treating SCC of the conjunctiva, it is suboptimal compared to SXRT. The surface sparing properties of electron beams can result in inadequate surface dose being delivered to the superficial target volume in SCC of the conjunctiva. In comparison, SXRT can be applied directly to the conjunctival surface, providing maximum surface dose, and limits the depth of penetration of harmful radiation. The comparatively deeper penetration of SXRT‐ in comparison to I131 and Sr90 plaque treatments‐ creates potential (albeit, minimal) for further treatment induced side effects. Whilst not present in this case study (at 6 months follow‐up), dose at depth‐ including the retina‐ has scope to induce radiation induced side effects pertinent to sight. Accurate dose recording to such deeper structures‐ with the use of thermoluminescent detectors (TLDs)‐ presents an excellent opportunity for future investigation in this patient cohort. Additionally, in this case, treatment with electron beam radiotherapy was not appropriate as the field size required to cover the target volume was less than the minimum field size able to be achieved with electron beam therapy. By using SXRT, the issue of field size with electron beam radiotherapy can be overcome, as the area irradiated can be confined to the small lesion size and spare adjacent healthy tissue. Thus, SXRT in comparison to electron beam treatment would provide better treatment coverage of the tumour volume and more adequately spare healthy tissue to preserve ocular function when treating recurrent SCC of the conjunctiva.

**Figure 5:**
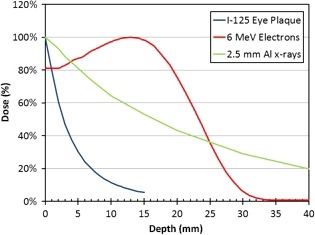
Graph comparing the depth penetration of Iodine plaque, 6 MeV electron & 2 mm Al superficial radiotherapy treatment (SXRT) (Graph courtesy of Division of Physical Sciences, Radiation Therapy, Peter MacCallum Cancer Centre).

SXRT may also be the preferred option compared to invasive treatments such as plaque therapy and enucleation. This case showed SXRT can be used as a salvage treatment alternative, avoiding the invasive alternative of orbital enucleation. The patient had previously undergone three separate excisions, plaque therapy and cryotherapy with subsequent recurrence after each treatment. There was potential for further plaque therapy treatment as an alternative option to SXRT in this case, given the low dose of 50 Gy in five fractions in the initial treatment. Supporting literature suggests that a dose of 100 Gy can be effective in reducing recurrence rates in these patients.^14^ However, SXRT was chosen in this case as a more penetrative treatment option was necessary given the measured depth of the tumour volume (2 mm). Figure 5 compares the doses at depth in millimetres for SXRT (2 mm Al); plaque therapy; and 6 MeV Electrons. This schematic demonstrates the percentage depth dose properties of SXRT, justifying it as the most appropriate option tumour volume coverage capability at depth (2 mm), whilst not compromising the required skin dose for a superficial lesion.

SXRT, in comparison to other radiotherapy techniques, offers more appropriate coverage of the tumour volume. However, it must be emphasised that effective treatment must consider limitations of size and location of the treatment area associated with SXRT. The field size for SXRT must provide coverage of the lesion with an acceptable margin to allow for random and systematic deviations in treatment delivery and microscopic disease in the periphery of the lesion. Our department limits the use of SXRT (with the Xstrahl© superficial unit) to lesions no smaller than 1.5 cm diameter, to ensure reliable dose uniformity across the treated area. In this case, we used a 2 cm diameter applicator direct approach. However, if the treated area required a lead shield to create an alternative field size (i.e. non‐circular to avoid healthy tissue), practical implications pertinent to shield position in relation to the eyelid retractors will need to be considered. An alternative method to ensure correct patient position may also be required. In accounting for these technical requirements, SXRT may be a successful treatment option for SCC of the conjunctiva.

These pertinent dosimetric benefits of SXRT are combined with improvements in operational efficiency. In comparison to other treatment options, SXRT does not require general anaesthesia or overnight hospital admission. The patient wore an eye shield for approximately 60 minutes after the procedure until the effect of the anaesthetic eye drops subsided. Conversely, SXRT does place a significant time burden on the patient (± carer(s)), with a requirement to attend twenty‐two consecutive treatment appointments (5/week). The procedure is not surgically invasive though the frequent attendance requirements may act as a deterrent for many considering SXRT. There are arguably departmental and therapeutic advantages to treating SCC of the conjunctiva with SXRT, though technical considerations need to be identified.

SXRT may be the preferred treatment option in cases of SCC of the conjunctiva if it can be proven to successfully manage the condition. As previously discussed, there is no evidence to support one treatment over the other for recurrent SCC of the conjunctiva. In this case, the lack of recurrence 6 months post‐treatment demonstrates a non‐inferior approach compared to previous interventions with improved cosmesis. As the ocular function of the left eye was maintained and visual acuity unaffected, this may have a positive effect on patient quality of life in comparison to other treatments for recurrent SCC of the conjunctiva, and although no measurement of quality of life was performed in this study, it would be of benefit in future investigations. Dry eye was the only reported side effect from treatment and is a common long‐term ocular side effect from radiotherapy treatment, occurring in 100% of cases when dose was in excess of 57 Gy.^15^ In this case, the patient was prescribed ocular lubricants to manage symptoms of dryness and monitored at follow‐up appointments. Dry eye was self‐reported and not graded pre or post‐treatment using subjective or objective grading scales. This was a limitation in this case study that may be included in further studies investigating SXRT for SCC of the conjunctiva.

## Conclusion

Follow‐up in this case study – evident by disease control at 6 months post‐treatment – provides a unique case to suggest SXRT as a viable option for the treatment of suitable cases of recurrent conjunctival SCC. Furthermore, in this instance, vision was not compromised whilst delivering an excellent cosmetic outcome. Currently, there is a distinct lack of evidence supporting the use of SXRT for SCC of the conjunctiva. This case study provides a snap shot of the role SXRT may be able to play as an effective treatment option for recurrent SCC of the bulbar conjunctiva. Further studies, in a larger patient cohort, are needed to further interrogate the role of SXRT as an alternative and less invasive treatment for SCC of the conjunctiva.

## Conflict of Interest

The authors declare no conflict of interest.
